# Gut microbiota, microbial metabolites, and mucosal barrier dysfunction in benign anorectal disorders and ulcerative colitis: shared mechanisms, clinical links, and precision interventions

**DOI:** 10.3389/fmed.2026.1979206

**Published:** 2026-10-12

**Authors:** Hexue Yuan, Maofang Zhao, Yunfen Wang, Chen Shen, Delong Liu, Chunxiao Wang, Jian Liu

**Affiliations:** 1 Department of Anorectal Surgery, The Gastrointestinal and Anorectal Hospital of Shenyang, Shenyang, Liaoning, China; 2 College of Chemical Science and Technology, Yunnan University, Kunming, China; 3 Department of Integrated Traditional Chinese and Western Medicine, Cardiology, The People's Hospital of Liaoning Province, Shenyang, China

**Keywords:** anal fistula, constipation, gut microbiota, hemorrhoidal disease, microbial metabolites, mucosal barrier, ulcerative colitis

## Abstract

Benign anorectal disorders and ulcerative colitis (UC) are typically managed as separate clinical conditions, despite growing recognition of overlapping pathophysiological features. Constipation, hemorrhoidal disease, anal fistula and colonic mucosal inflammation may converge on partially shared molecular pathways involving microbial dysbiosis, altered microbial metabolite generation, epithelial barrier impairment, immune activation, stromal remodeling and dysregulated tissue repair, although the strength of mechanistic evidence varies substantially among these conditions. This review synthesizes advances from the past decade to propose a translational molecular medicine framework for investigating potential molecular connections among anatomically distinct anorectal and colonic disorders, without assuming shared causal origins. Clinical and pathological associations between disordered defecation, hemorrhoidal vascular remodeling, fistula persistence and UC are contextualized alongside current insights into gut and anorectal microbiota dysbiosis, short-chain fatty acid signaling, bile acid metabolism, tryptophan metabolite activity, gas-producing microbial communities, mucus biology, tight junction function, innate and adaptive immune responses, and fibro-inflammatory repair processes. Integrating microbiome profiling, metabolomics, transcriptomics, single-cell and spatial analyses with causal inference provides a cross-disease framework for resolving convergent molecular states associated with recurrence, healing and therapeutic response. By moving beyond disease-by-disease description, this strategy may uncover biomarkers and actionable pathways otherwise overlooked, supporting mechanism-informed prevention, perioperative optimization and individualized intervention across anorectal disorders and inflammatory bowel disease (IBD).

## 1 Introduction

Benign anorectal disorders and ulcerative colitis (UC) represent frequent indications for consultation across colorectal surgery, gastroenterology and primary care settings. Clinical management of hemorrhoidal disease, anal fistula, anal fissure and constipation-related anorectal symptoms is traditionally guided by anatomical location, symptom severity and procedural eligibility, while UC is classified and treated as a chronic relapsing inflammatory bowel disorder (1–4). This compartmentalized clinical model offers practical utility for routine care, yet may obscure underlying biological connections operating at the interface between luminal contents, the anorectal mucosa, resident microbial communities, epithelial barrier function and local immune responses (5, 6). Contemporary clinical guidelines provide increasingly refined management algorithms for hemorrhoidal disease, anorectal abscess and fistula, chronic idiopathic constipation and UC, while simultaneously highlighting persistent unmet needs in recurrence prevention, addressing symptom heterogeneity and delivering individualized therapeutic strategies (1–4, 7). The molecular basis underpinning these clinical limitations remains incompletely characterized.

Culture-independent microbiology, metabolomics, immune profiling, and tissue-resolved methods have reshaped intestinal pathobiology over the past decade. Large inflammatory bowel disease (IBD) multi-omics cohorts show coordinated shifts in microbial composition, microbial transcription, fecal metabolites, and host inflammation across activity states (5, 8). Functional work in constipation indicates that fermentation products, methane, and related metabolites can influence transit, epithelial secretion, visceral sensation, and mucosal immune tone (9, 10). In anal fistula and anorectal abscess, 16S rRNA sequencing and source-tracking across tracts, perianal skin, rectal mucosa, abscess cavities, and feces challenge a purely classical cryptoglandular account of infection (11, 12). Molecular evidence in hemorrhoidal disease is thinner, although epidemiology and Mendelian randomization justify more systematic study of host susceptibility, inflammation, bowel function, and microbial exposure (13–15).

The present review treats constipation, hemorrhoidal disease, anal fistula, and UC as clinically distinct entities that may nonetheless engage overlapping molecular modules. These modules include microbial dysbiosis, altered metabolite signaling, epithelial barrier stress, innate and adaptive immune activation, stromal remodeling, and impaired tissue repair (5, 6, 16). Shared modules are invoked to enable comparative mechanistic analysis, not to collapse etiology. Hemorrhoidal disease is not assumed to belong within the IBD spectrum, fistula pathogenesis is not assumed to be universally microbiota-dependent, and constipation and UC are not treated as poles of one continuum. Distinct phenotypes may instead reflect conserved processes acting in different anatomical settings, with different triggers, intensities, and time courses. That distinction limits overgeneralization while permitting mechanistic integration (6, 17).

Accordingly, this review advances a cross-disease translational framework rather than simply juxtaposing evidence from constipation, hemorrhoidal disease, anal fistula, and UC. We propose that these anatomically and clinically distinct disorders may converge on partially shared biological modules involving microbial ecosystem disturbance, altered metabolite signaling, mucosal barrier dysfunction, immune–stromal remodeling, and defective tissue repair, while differing substantially in the relative importance, anatomical distribution, and temporal evolution of these processes. This framework integrates clinical and pathological observations with site-resolved microbiome evidence and molecular mechanisms, and considers how longitudinal sampling, multiomics integration, and causal validation could identify recurrence-associated signatures, mechanistically informative biomarkers, and actionable pathways that may remain obscured when each disorder is studied in isolation. The objective is not to imply shared etiology or biological equivalence, but to establish a testable basis for cross-disease molecular comparison and mechanism-informed precision intervention.

This article is a narrative, non-systematic review. Relevant literature was identified through targeted searches using combinations of disease-specific terms and concepts related to microbiota, microbial metabolites, mucosal barrier dysfunction, immune-stromal remodeling, tissue repair, multiomics, and precision medicine. Priority was given to recent peer-reviewed studies, clinical guidelines, high-quality reviews, human observational and interventional studies, and mechanistically informative experimental work. The evidence was synthesized thematically to distinguish shared biological modules from disease-specific mechanisms and to highlight areas of differing evidence maturity. Because this was not a systematic review, no formal protocol, duplicate screening, risk-of-bias assessment, or meta-analytic synthesis was undertaken.

## 2 Clinical links and shared pathological basis

Clinical links between benign anorectal disorders and UC are closely tied to defecatory behavior, the luminal environment, and host tissue responses. Stool frequency, consistency, straining, urgency, tenesmus, and incomplete evacuation jointly shape mechanical and microbial conditions in the anorectum. Constipation can prolong stool residence, promote luminal dehydration, and raise expulsive pressure, thereby increasing stress on the anal cushions and distal rectal mucosa (1, 4, 13). These forces may contribute to mucosal injury, vascular congestion, connective-tissue remodeling, and persistent hemorrhoidal symptoms. After hemorrhoid or fistula procedures, ongoing constipation may impede evacuation, load the wound, and delay recovery (18, 19). Constipation is therefore both a symptom and a potentially modifiable determinant of anorectal disease course and recurrence.

Beyond mechanics, constipation can remodel the intestinal microenvironment through altered transit, substrate availability, luminal pH, microbial exposure, and metabolite output (9, 10). Studies in functional constipation report shifts involving Bifidobacterium, Lactobacillus, Bacteroides, Prevotella, and butyrate-associated taxa, although results vary with population, diet, sequencing methods, and constipation phenotype (9). Methanogenic archaea have drawn attention because methane production associates with delayed transit, yet links to symptom severity remain inconsistent (10). The interaction is bidirectional. Transit conditions reshape microbial ecology, while microbial signals may further influence motility, secretion, and visceral sensation.

Hemorrhoidal disease has long been explained by cushion descent, venous enlargement, connective-tissue degeneration, and abnormal defecatory forces (1, 13). These structural accounts do not fully explain recurrent inflammation, thrombosis, bleeding, or persistent symptoms after intervention. Local inflammatory responses, angiogenic activity, extracellular matrix remodeling, and vascular permeability may contribute to clinical expression (1). Continuous exposure of anorectal tissues to luminal microbes and metabolites provides a plausible rationale for studying microbiota-associated inflammation and impaired tissue homeostasis, although such mechanisms remain largely hypothetical and require experimental testing (14, 15).

Anal fistula sits at a more direct interface of microbial exposure, immune activation, and defective repair. Cryptoglandular obstruction, infection, and abscess formation explain many cases, yet they do not fully clarify why some lesions resolve while others evolve into chronic tracts or recur after surgery (2, 16). Persistence likely reflects coordinated interactions among local communities, tract epithelialization, matrix remodeling, immune responses, and ongoing fecal contamination (11, 12, 16). Effective care therefore requires infection control together with restoration of tissue integrity and preservation of anorectal function.

UC, by contrast, is a prototypical immune-mediated mucosal disease marked by epithelial injury, barrier disruption, altered mucus organization, inflammatory infiltration, and recurrent immune activation (3, 5, 8). Fistulizing disease is not a defining UC feature, yet patients may experience constipation-like symptoms, urgency, tenesmus, perianal discomfort, and functional anorectal abnormalities related to inflammation, motility change, pelvic-floor dysfunction, or coexisting anorectal pathology (20, 21). Assessment must therefore separate active inflammation from functional disorders and structural anorectal disease.

Figure 1 proposes a module-based reading of these clinically distinct conditions. Rather than implying one shared pathway, it highlights convergence on interacting domains that include microbial ecosystem instability, metabolite-mediated signaling, epithelial barrier stress, and immune-stromal remodeling. Relative weights differ by disease. Constipation and hemorrhoidal disease are strongly shaped by stool properties, mechanical force, and local tissue remodeling; anal fistula is dominated by persistent microbial exposure and failed repair; UC is driven chiefly by sustained epithelial injury and dysregulated mucosal immunity. Acknowledging these differences allows shared molecular processes to inform comparative research without erasing disease-specific biology.

**Figure 1:**
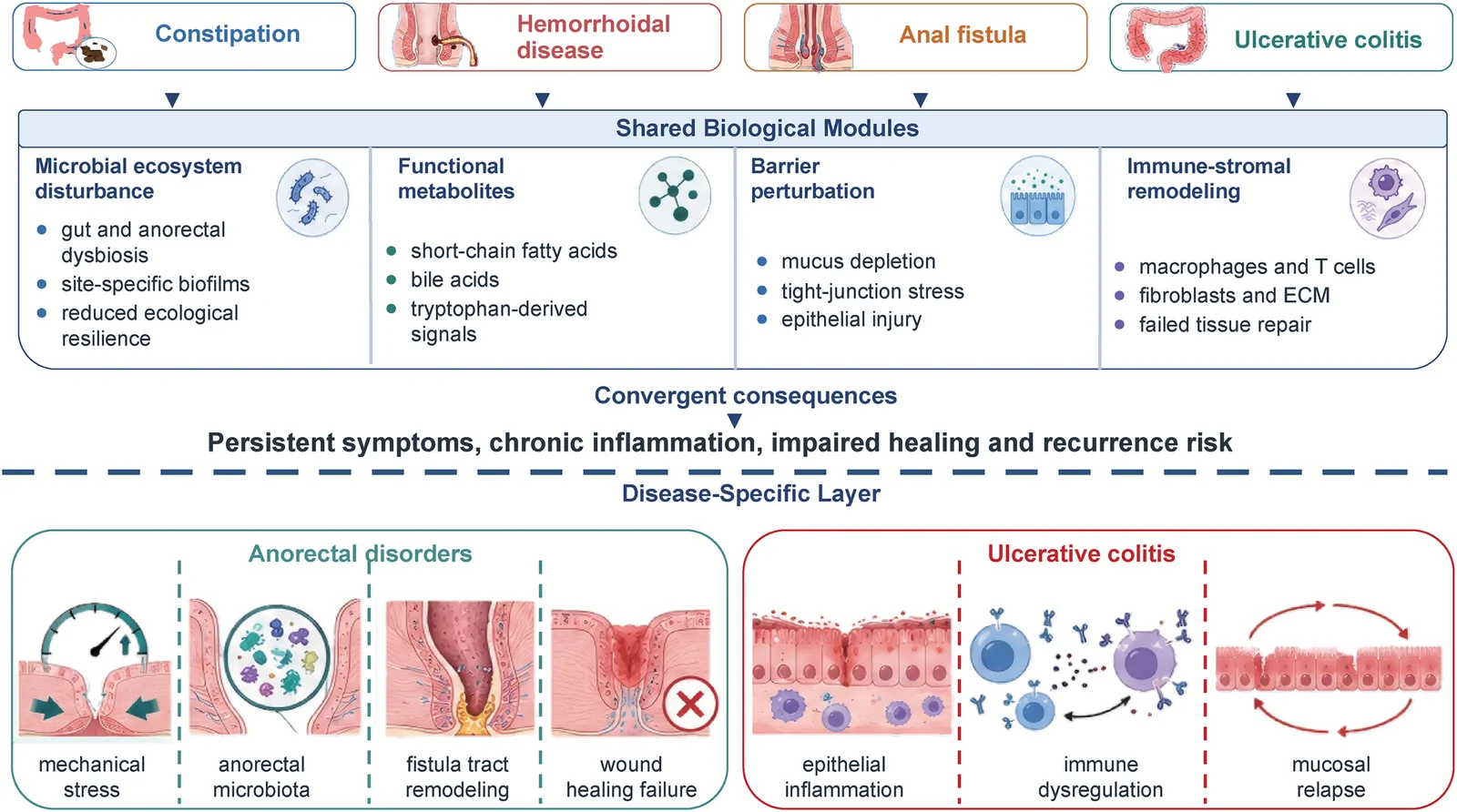
Shared molecular landscape connecting benign anorectal disorders and UC. The diagram is organized into three levels. The upper row shows the four clinical phenotypes considered in this review. The central region depicts the major molecular domains used for cross-disease comparison and their convergence on persistent inflammation, impaired healing and recurrence. The lower region separates anorectal disorders from UC to indicate the dominant disease-specific determinants operating within each context. Solid arrows indicate the proposed direction of biological interaction rather than proven linear causality. Color coding denotes microbial processes, metabolite signaling, epithelial barrier biology and immune-stromal remodeling. ECM, extracellular matrix; SCFAs, short-chain fatty acids; UC, ulcerative colitis.

Evidence maturity remains uneven. UC is supported by extensive multi-omics, single-cell, and biomarker datasets, whereas constipation has emerging but less definitive microbiota-motility evidence. Fistula research increasingly addresses microbial ecology, stromal remodeling, and regenerative strategies, while hemorrhoidal disease remains comparatively underexplored at the molecular level (5, 8, 11–16). Table 1 summarizes major disease contexts, shared molecular features, disease-specific components, evidence maturity, and priority translational questions.

**Table 1:** Shared and disease-specific molecular features across anorectal disorders and UC.

Disease context	Shared molecular features	Disease-specific features	Evidence maturity	Priority questions
Constipation	Transit-microbiota feedback, fermentation imbalance, methane-associated motility changes, barrier stress from stool stasis	Straining, hard stool and altered pelvic floor dynamics dominate the phenotype	Moderate for microbiota association; limited mechanistic validation	Define molecularly distinct constipation subtypes and test whether microbiota-targeted therapy changes outcomes
Hemorrhoidal disease	Low-grade inflammation, dysbiosis signals, barrier microtrauma, vascular and connective-tissue remodeling	Venous congestion, prolapse biology and local mechanical stress are central	Strong clinical literature; weak molecular evidence	Build tissue and anorectal swab studies linked to symptom patterns, recurrence and repair
Anal fistula	Mixed microbial communities, chronic inflammation, fibroblast activation, ECM remodeling and failed repair	Crypt infection, abscess evolution and tract epithelialization are distinctive	Emerging local microbiome and tissue-level evidence	Integrate tract microbiota with histology, spatial transcriptomics and healing trajectories
UC	Mucosal dysbiosis, altered metabolites, barrier dysfunction and amplified immune signaling	Continuous colonic inflammation and relapse-prone mucosal disease define the phenotype	Strong mechanistic evidence from human and experimental studies	Refine endotypes that explain treatment response, relapse and extraintestinal variability

## 3 Gut microbiota dysbiosis across anorectal and colonic disorders

Microbiota dysbiosis is better framed as a functional disturbance than as a catalogue of taxon shifts. In UC, active disease commonly features reduced alpha diversity, loss of obligate anaerobes and butyrate-producing Firmicutes, expansion of facultative taxa, altered mucin-related communities, and heightened community instability (22, 23). Taxonomic lists remain insufficient because strains within one genus can diverge in metabolism and immune effects. Integrated multi-omics therefore emphasize microbial gene content, transcriptional activity, metabolite output, and host response. Disease activity tracks with altered microbial transcription, short-chain fatty acid depletion, disrupted bile acid conversion, oxidative stress-related pathways, and mucosal immune activation, thereby strengthening the microbiota-metabolite-barrier axis (5, 22–25).

Constipation microbiome findings are far less consistent. Reports variously implicate reduced fermentative taxa, shifts in Bifidobacterium or Lactobacillus, Bacteroidetes remodeling, methane-producing archaea, or mucin-associated lineages (9, 10). Heterogeneity likely arises from divergent case definitions, fiber intake, medications, age, stool form, sequencing depth, and the mix of slow-transit constipation, defecatory disorders, IBS-C, and chronic idiopathic constipation. The central conceptual advance is bidirectionality between transit and the microbiome. Prolonged retention reshapes fermentation niches, whereas microbial metabolites can modulate epithelial secretion, enteroendocrine signaling, enteric nerves, and smooth-muscle activity (9, 10, 26). This reciprocity blurs causality yet widens therapeutic leverage.

Anorectal niches should not be equated with stool. The anal canal and distal rectum experience stool exposure together with unique epithelial transitions, oxygen gradients, local secretions, cutaneous contact, hygiene practices, topical agents, and surgical wounds. Recent 16S rRNA sequencing and source-tracking in anal fistula and anorectal abscess show that lesion communities may derive from intestinal contents as well as perianal skin, and that sites within one patient can diverge sharply (11, 12, 27). Fecal sampling alone may therefore miss organisms relevant to recurrence within residual tracts, abscess cavities, internal openings, or external wounds. For cryptoglandular abscess and fistula, classical culture described mixed aerobic-anaerobic infection, whereas culture-independent work now resolves anaerobes, facultative pathogens, and site-linked signatures potentially tied to inflammation, biofilm-like persistence, or impaired healing (12, 27, 28). The field remains early. Most cohorts are small, cross-sectional, and geographically narrow, and few standardize sampling across the internal opening, tract, rectal mucosa, perianal skin, and stool. Antibiotics, drainage timing, prior surgery, incomplete Crohn exclusion, and wound status further confound inference. Outcome-linked designs that capture recurrence, fistula healing, postoperative pain, and continence are still needed.

Hemorrhoidal disease has the sparsest microbiome evidence among anorectal disorders. Recent studies have suggested a potential relationship between microbiota alterations and hemorrhoidal pathophysiology. Palumbo et al. proposed that altered anal canal microflora may contribute to local inflammation and anorectal dysfunction, although this hypothesis requires further validation (29). Yang et al. reported potential causal associations between gut microbial taxa and hemorrhoidal disease using Mendelian randomization analysis, but these findings mainly reflect gut-level associations rather than local microbial changes within hemorrhoidal tissues (14). Wang et al. further demonstrated distinct microbiomic characteristics of hemorrhoids compared with fecal and rectal surface microbiota, suggesting that local microbial niches may involve both intestinal and skin-associated sources (30). Stronger inference will require paired fecal, anorectal swab, and tissue sampling aligned with grade, bleeding activity, constipation burden, defecatory mechanics, and histology. Until then, microbiota are better considered candidate modifiers rather than confirmed drivers of hemorrhoidal disease.

UC research shows that dysbiosis is dynamic across inflammation, medication, diet, and remission (5, 22, 23). Analogous state dependence is plausible in anorectal disease, for example across drainage, seton placement, definitive closure, and recurrence, or between acute thrombosis and chronic prolapse. Constipation communities may likewise shift after fiber, osmotic agents, secretagogues, probiotics, or pelvic-floor therapy. Longitudinal sampling is therefore essential to separate drivers from sequelae and to identify molecular states that predict recurrence or treatment response.

Translational value hinges on reproducibility and clinical actionability. Classifiers trained on small cohorts often fail externally, and machine-learning analyses must control leakage, overfitting, and batch effects with independent validation (31, 32). For anorectal disorders, useful tools would forecast fistula recurrence risk, slow wound healing, microbiota-targeted constipation response, or UC endotypes defined by metabolite deficiency or barrier impairment. Table 2 summarizes microbiota-related mechanisms and research priorities across the four disorders.

**Table 2:** Microbiota-associated mechanisms and sampling priorities across disease contexts.

Disease context	Priority sample types	Representative observations	Key confounders	Best next-step design
Constipation	Feces, diet records, breath methane, transit measurements	Reduced fermentative efficiency or altered methanogen signatures in some cohorts; links to motility remain heterogeneous	Fiber intake, laxatives, stool consistency and functional subtype	Longitudinal sampling before and after diet, laxative or motility-directed intervention
Hemorrhoidal disease	Feces, anorectal swabs, hemorrhoidal tissue, symptom scores	Very limited data suggest local inflammation and possible dysbiosis contributions	Bleeding, hygiene, topical therapy, grade and thrombosis status	Case-control and recurrence cohorts with paired stool and local tissue profiling
Anal fistula and abscess	Tract tissue, internal opening swabs, abscess fluid, paired stool	Site-specific communities, chronic inflammatory biofilms and healing-associated divergence are plausible	Antibiotics, drainage timing, prior surgery and disease complexity	Paired site sampling linked to MRI, surgery and recurrence outcomes
UC	Mucosal biopsies, stool, serum metabolites, endoscopy and histology	Reduced microbial diversity, metabolite disruption and barrier-associated inflammatory signatures are recurrent	Medication exposure, diet, disease extent and activity state	Prospective endoscopy-linked cohorts integrating mucosal omics with relapse and treatment response

## 4 Microbial metabolites, mucosal barrier dysfunction, and immune activation

Microbial metabolites form a mechanistic bridge between community structure and host biology. Short-chain fatty acids generated by fermentation of dietary fibers and resistant starches supply colonocyte energy and regulate epithelial oxygen consumption, junctional integrity, mucus output, and anti-inflammatory programs (33–35). Signaling through G-protein-coupled receptors and histone deacetylase inhibition further shapes regulatory T cells, macrophages, and epithelial repair. In UC, reduced butyrate production and impaired epithelial utilization accompany inflammation, although causal hierarchy remains context dependent (33–35). In constipation, SCFA effects on motility and secretion vary with concentration, intestinal segment, and host sensitivity. Distal rectal and anal exposure to fecal SCFAs is poorly quantified, so local uptake and wound-related actions remain speculative. Adequate butyrate tone may favor barrier maintenance, whereas dysbiosis, pH shift, or epithelial injury could weaken metabolic support and sustain neutrophil influx, protease release, and matrix breakdown in fistula wounds. These possibilities require longitudinal metabolomics of wound fluid, tissue, and stool (34, 35).

Bile acids provide a second major metabolite class. Microbial conversion of primary acids yields secondary species that engage farnesoid X receptor and Takeda G-protein-coupled receptor 5, thereby influencing secretion, immunity, barrier function, microbial ecology, and motility (24, 36–38). In IBD, altered bile acid pools and reduced secondary conversion track with inflammation and dysbiosis (24, 36, 37). In constipation, colonic bile acids promote secretion and transit, and ileal bile acid transporter inhibition is already used therapeutically, positioning bile acids as biomarkers and mediators across the motility-inflammation interface (38, 39).

Tryptophan metabolism links ecology to mucosal immunity through indole ligands of the aryl hydrocarbon receptor. Microbial AhR agonists support barrier defenses, antimicrobial peptides, and IL-22-dependent mucosal protection (40, 41). Impaired microbial tryptophan metabolism associates with inflammation in IBD models, and restoring AhR signaling improves barrier responses (40, 41). Direct anorectal data are scarce, yet the pathway is biologically pertinent because the distal gut depends on antimicrobial peptides, epithelial turnover, and immune tolerance under constant microbial load. Local shortage of protective indoles could therefore hinder wound healing or amplify inflammation.

Gas and redox metabolites add further complexity. Methane associates with slower transit and constipation-predominant phenotypes, whereas hydrogen sulfide shows concentration-dependent actions ranging from signaling to epithelial toxicity (42, 43). Nitric oxide, reactive oxygen species, and sulfur compounds may also affect vascular tone, epithelial stress, and immune activation. These species are hard to quantify and vary along the gut, yet they are especially relevant where mechanical stress, vascular congestion, and bacterial exposure converge in the anorectum. Integrated breath testing, fecal metabolomics, and local tissue markers are preferable to taxon-only inference (42, 43).

On the host side, mucosal barrier failure is the counterpart of metabolite disruption. Mucus, epithelium, junctional complexes, antimicrobial peptides, secretory IgA, epithelial renewal, and immune surveillance normally limit antigen access (44, 45). UC features goblet-cell dysfunction, mucus loss, ulceration, and increased permeability that allow microbial products to activate lamina propria immunity. Anorectal surfaces are likewise vulnerable to hard stool, straining, diarrhea, urgency, topical irritation, and surgical trauma; incomplete repair permits ongoing contamination, inflammation, and pain. Claudins, occludin, and zonula occludens proteins govern paracellular flux and respond to cytokines as well as microbial products and metabolites. TNF, IL-6, IL-13, IL-17, and interferon-related pathways can remodel junctions and epithelial turnover, whereas SCFAs and AhR ligands may offset injury through energy support, mucus defense, and anti-inflammatory signaling (44, 45). Compositional dysbiosis therefore matters clinically mainly when it alters metabolites, epithelial function, or immune tone.

Innate activation spans a spectrum from low-grade local irritation to chronic mucosal inflammation. Neutrophil and macrophage programs dominate active UC and also participate in abscess formation, tract persistence, and repair in anal fistula, where ongoing bacterial exposure can maintain cytokine gradients and protease activity. In hemorrhoidal disease, local mediators may intensify bleeding, edema, and pain, whereas constipation-related immune signals remain inconsistent and often secondary to barrier stress or luminal retention. Tissue repair then determines outcome. Controlled inflammation, epithelial restitution, angiogenesis, and matrix remodeling restore homeostasis, but unresolved inflammation can establish a fibro-inflammatory niche (16). Epithelial-to-mesenchymal transition-like programs, matrix metalloproteinases, and stromal activation are implicated in Crohn fistula persistence and may operate, though less mapped, in cryptoglandular disease. Connective-tissue degeneration and vascular remodeling dominate hemorrhoidal structure, while repeated injury-repair cycles in UC reshape mucosa and, in some patients, contribute to architectural distortion or dysplasia risk.

Figure 2 summarizes metabolite-barrier-immune interactions. Metabolite actions switch between protection and injury according to concentration, anatomical niche, and host state. Barrier failure can initiate as well as amplify immune responses rather than merely following them. Immune and stromal programs then decide whether tissue returns to homeostasis, scars, remains inflamed, or recurs after treatment. Table 3 lists major microbial metabolites and candidate mechanisms in anorectal and colonic disease.

**Figure 2:**
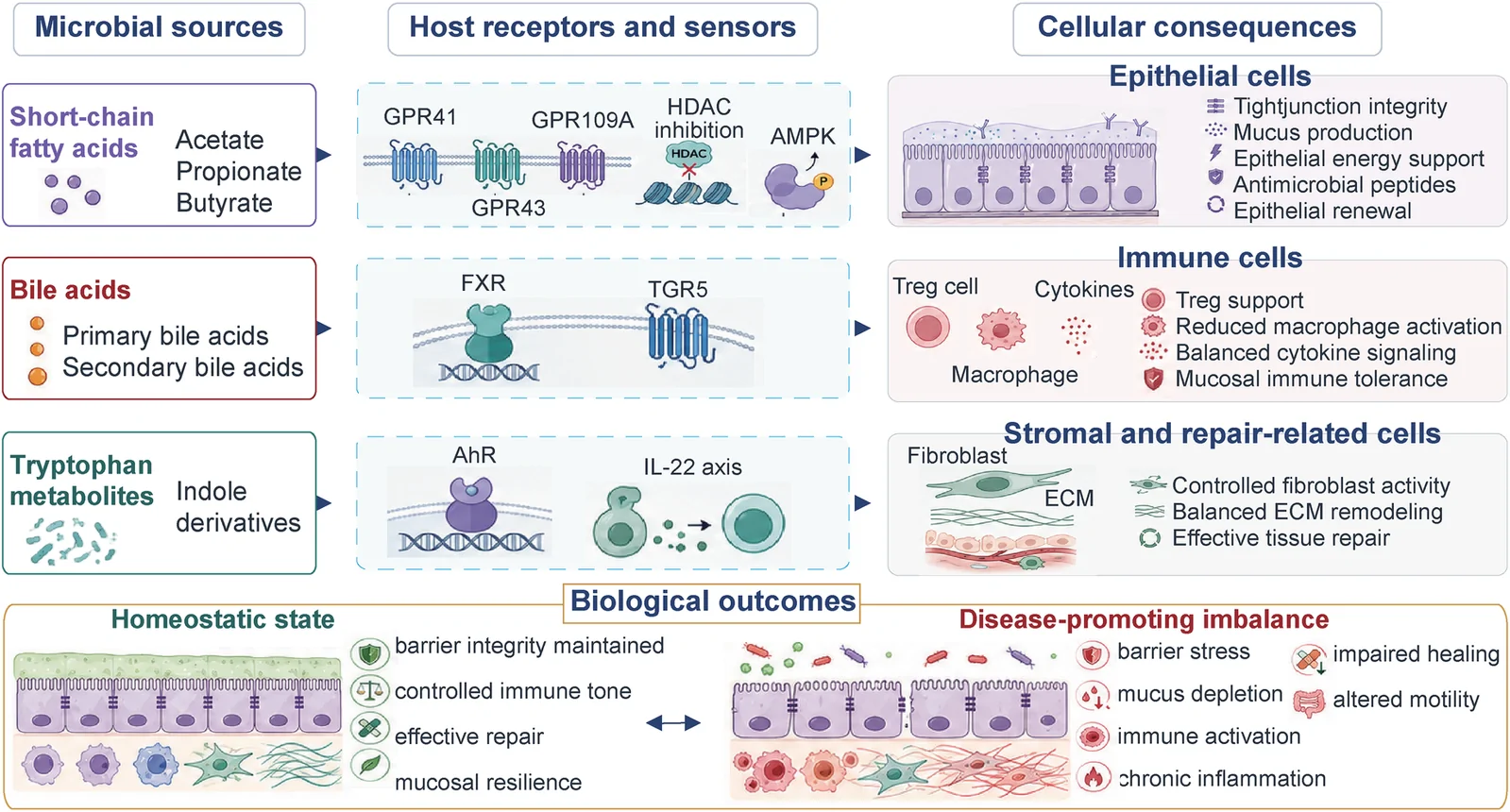
Microbial metabolite signaling controls epithelial barrier integrity and immune homeostasis. The left column shows three major classes of microbial functional outputs, namely short-chain fatty acids, bile acids and tryptophan-derived metabolites. The central column maps these signals to major host receptors and sensors, including GPR41, GPR43, GPR109A, FXR, TGR5 and AhR. The right column summarizes representative cellular consequences in epithelial, immune and stromal compartments, including support of tight-junction integrity, mucin production, ion transport, antimicrobial defense, Treg-associated signaling and balanced inflammatory tone. The bottom banner summarizes the net biological message that protective metabolite signaling reinforces barrier and immune homeostasis, whereas disturbed metabolite pools may contribute to motility abnormalities, barrier stress and chronic inflammation. AhR, aryl hydrocarbon receptor; AMPK, AMP-activated protein kinase; FXR, farnesoid X receptor; HDAC, histone deacetylase; SCFAs, short-chain fatty acids; TGR5, G protein-coupled bile acid receptor 1.

**Table 3:** Microbial metabolites, host receptors and barrier-immune effects.

Metabolite class	Major origin	Key host receptors or sensors	Barrier and immune effects	Disease relevance
Short-chain fatty acids	Fiber fermentation by anaerobic commensals	GPR41, GPR43, GPR109A, HDAC inhibition, AMPK	Support epithelial energy metabolism, mucus production, tight junctions and Treg-associated immune restraint	Likely protective in UC; may also influence constipation biology and postoperative repair
Bile acids	Host synthesis with microbial deconjugation and transformation	FXR, TGR5 and epithelial secretory programs	Shape motility, secretion, innate responses and microbial ecosystem structure	Relevant to constipation, dysmotility states and inflammation-linked microbiota shifts
Tryptophan metabolites	Microbial indoles and host kynurenine pathways	AhR, IL-22 axis and epithelial transcriptional programs	Promote antimicrobial peptide expression, epithelial renewal and controlled mucosal immunity	Strongest evidence in UC, but conceptually relevant to anorectal wound healing and tolerance
Potentially harmful metabolites	Hydrogen sulfide, oxidative products and context-dependent amines	Cell stress pathways, mitochondrial dysfunction and inflammatory signaling	May impair barrier integrity, worsen epithelial stress and amplify inflammation	Requires careful site-specific study because context and concentration determine pathogenicity
Lipid mediators and related metabolites	Microbiota-host co-metabolism of lipids and phospholipids	PPAR-linked signaling and membrane remodeling responses	Can influence stromal activation, inflammation resolution and tissue repair tone	An emerging area with possible relevance to fistula persistence and mucosal relapse

## 5 Multiomics, causal inference, and precision stratification

A central limitation of current evidence is the gap between association and causality. Taxonomic differences between patients and controls may reflect diet, medications, inflammation, transit time, stool consistency, antibiotics, bowel preparation, or sampling site rather than disease drivers. Separating driver, mediator, and consequence therefore requires designs with temporal or experimental leverage. Longitudinal cohorts can test whether microbial or metabolite shifts precede symptoms, flare, or recurrence, whereas intervention trials ask whether microbiota change alters clinical outcomes. Mendelian randomization may lessen confounding when genetic instruments are valid, yet it cannot replace mechanistic experiments (14, 26, 46). Germ-free and gnotobiotic animals, organoids, epithelial co-cultures, and tissue explants remain essential for testing whether candidate microbes or metabolites affect barrier repair or immune activation (17, 47).

Multiomics is valuable because each layer captures a distinct biological scale. Amplicon sequencing offers taxonomic breadth with limited strain and functional detail, whereas shotgun metagenomics improves resolution and pathway inference. Metatranscriptomics reveals active microbial genes, metabolomics quantifies host-facing outputs, and host transcriptomics maps epithelial, immune, and stromal programs. Single-cell profiling resolves disease-associated cell states, and spatial transcriptomics retains anatomical context that bulk assays erase (5, 8, 22, 23, 48, 49). For anorectal disease, spatial resolution is indispensable because internal openings, fistula tracts, external wounds, hemorrhoidal mucosa, and perianal skin are non-equivalent niches. Tissue-resolved UC studies already illustrate this power. Single-cell atlases show that inflammation rewires epithelial, stromal, and immune compartments and restructures cellular communication rather than merely expanding leukocyte numbers (8, 48, 49). Analogous mapping could clarify whether persistent fistula tracts harbor specialized epithelial-like cells, activated fibroblast subsets, wound-associated macrophages, distinct neutrophil states, or spatially constrained microbial niches. Figure 3 frames this continuum from crypt injury and infection through acute inflammation, chronic niche formation, and fibro-inflammatory remodeling to failed regeneration, and indicates where infection control, immune modulation, and regeneration-oriented strategies may act along that trajectory.

**Figure 3:**
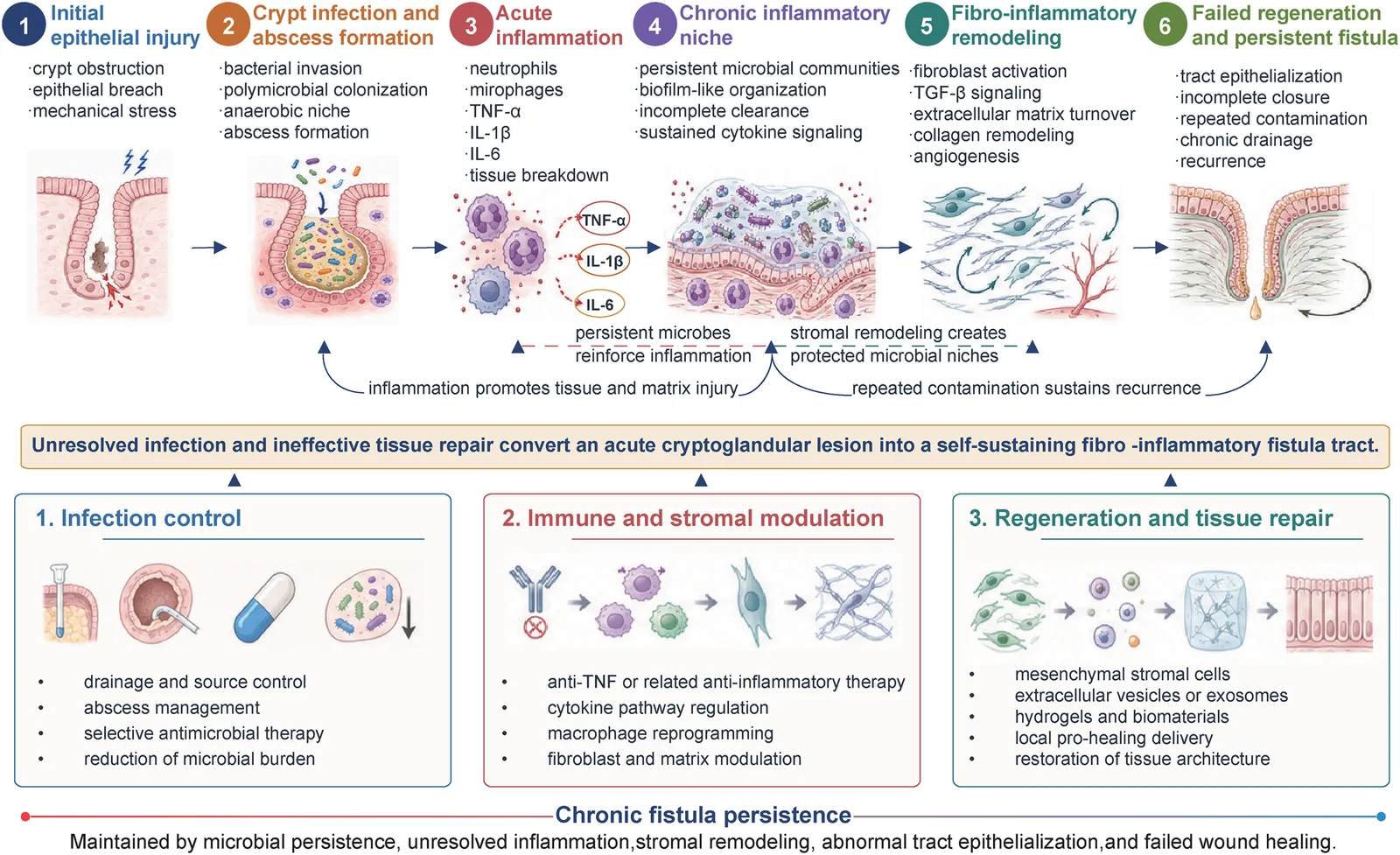
Molecular mechanisms driving chronic anal fistula formation and recurrence. The upper trajectory depicts a staged transition from initial injury and crypt infection to acute inflammation, chronic inflammatory niche formation, fibro-inflammatory remodeling and failed regeneration. The central banner emphasizes the pathogenic transition in which unresolved infection and ineffective repair convert an acute lesion into a self-sustaining fistula tract. The lower tier identifies three therapeutic windows across the continuum, including infection control, immune modulation and regeneration-focused repair. Together, the figure frames chronic fistula not simply as an anatomical tract but as a dynamic interaction among microbes, inflammation, stromal remodeling and incomplete healing. ECM, extracellular matrix; TNF, tumor necrosis factor.

In constipation and hemorrhoidal disease, multiomics may first refine phenotyping rather than isolate single causal pathways. Slow-transit constipation, defecatory disorder, IBS-C, and medication-related or systemic secondary constipation likely diverge in microbiota, metabolites, neuromuscular function, and inflammatory tone. Hemorrhoidal phenotypes likewise differ by bleeding, prolapse, thrombosis, pain, and post-treatment recurrence. Integrating symptoms, anorectal physiology, stool form, diet, microbiota, and local tissue markers may identify subgroups responsive to fiber strategies, microbiota modulation, pelvic-floor therapy, or selected procedures.

Precision stratification depends on harmonized sampling and analytical rigor. Fecal studies should capture stool consistency, bowel preparation, laxatives, antibiotics, probiotics, diet, disease activity, surgery, and freezing delay. Anorectal swabs require standardized depth, contact time, skin contamination control, and timing relative to cleansing or procedures. Fistula work should separately annotate internal opening, tract material, abscess fluid, rectal mucosa, perianal skin, and stool. UC protocols should record inflamed versus noninflamed mucosa, rectal versus proximal sites, and medication exposure. Without such harmonization, signatures risk tracking technical artifact (50). Batch correction, compositional methods, false-discovery control, pre-specified endpoints, and independent validation are therefore mandatory (29, 51). Machine-learning models require separate training and test cohorts plus assessment of calibration, discrimination, and clinical utility; a high area under the curve in a small single-center set is not translationally sufficient (31, 32). Interpretable models that point to metabolite-deficient or barrier-impaired biology can guide fiber optimization, targeted probiotics, butyrate-related approaches, or deferred definitive surgery until inflammation is controlled.

Figure 4 outlines a precision roadmap from prospective cohorts and harmonized multidimensional sampling through integrated multiomics and computational modeling to biologically informed stratification. The lower panel proposes conceptual molecular states centered on microbiota dominance, barrier failure, immune-inflammatory activity, and fibro-remodeling. Although still hypothetical, these endotypes offer a more mechanistic scaffold than symptom-only labels and a basis for matching diet optimization, microbiota-directed therapy, metabolite replacement, anti-inflammatory treatment, or repair-focused intervention. In that sense, Figure 4 is a translational hypothesis about how molecular profiling may reshape clinical decisions. Table 4 summarizes multiomics platforms and precision intervention opportunities.

**Figure 4:**
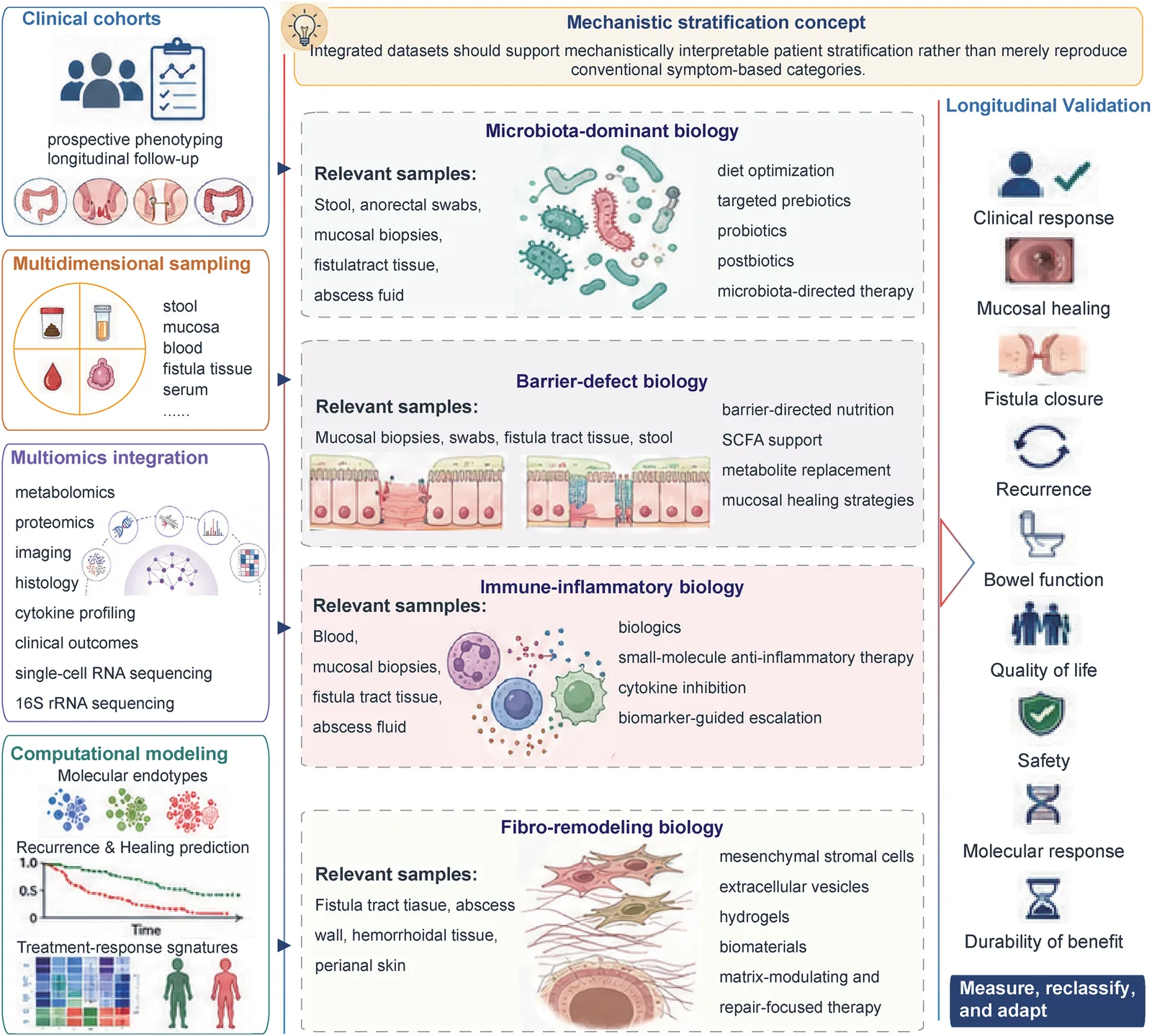
Multiomics-driven precision medicine framework for anorectal disorders and UC. The upper workflow proceeds from prospective clinical cohorts and multidimensional biospecimen collection to integrated multiomics and computational modeling. The central banner underscores that integrated datasets can support biologically informed stratification rather than symptom-only classification. The lower panel then proposes four partially overlapping conceptual molecular states, including microbiota-dominant, barrier-defect, immune-inflammatory and fibro-remodeling states, each linked to representative candidate features and intervention directions. The figure is intended as a translational roadmap for future studies rather than as a finalized clinical algorithm. FMT, fecal microbiota transplantation; MSC, mesenchymal stromal cell.

**Table 4:** Multiomics platforms and precision intervention opportunities.

Platform	Best suited specimens	Principal output	Precision medicine use	Main limitation
16S rRNA sequencing	Stool, swabs and tract samples	Broad bacterial community structure	Initial dysbiosis mapping and low-cost cohort stratification	Limited strain and functional resolution
Shotgun metagenomics	Stool and mucosal samples	Species, strains and metabolic pathways	Functional microbiome endotyping and pathway discovery	Cost, host DNA contamination and analytical complexity
Metabolomics	Stool, serum, tissue and wound fluid	SCFAs, bile acids, indoles and pathway activity readouts	Biomarker discovery and targetable metabolic deficits	High sensitivity to diet, storage and sampling workflow
Bulk transcriptomics	Mucosal biopsies and tissue specimens	Inflammatory, barrier and repair programs	Contextualize host responses and molecular endotypes	Cell-type averaging can obscure critical niches
Single-cell and spatial transcriptomics	Biopsies, hemorrhoidal tissue and fistula tracts	Cell states, immune-stromal niches and spatial organization	Identify reparative versus pathogenic cellular programs	Cost, sample handling constraints and modest throughput
Integrated machine learning models	Clinical, imaging and multiomics datasets	Subtype assignment, relapse prediction and treatment response signatures	Translate complex data into actionable precision stratification	Requires large harmonized cohorts and external validation

## 6 Therapeutic implications, evidence gaps, and translational roadmap

Mechanism-informed intervention should complement rather than replace established clinical management. Fiber intake, hydration, physical activity, appropriate toilet behavior, and control of excessive straining remain foundational for constipation and hemorrhoidal disease and retain perioperative relevance (4, 7, 52). Fiber also provides substrate for microbial fermentation and short-chain fatty acid production, although its effects depend on chemical properties, dose, and tolerance. Rapidly fermentable fibers may aggravate bloating, whereas poorly fermentable or gel-forming fibers can improve stool form with fewer symptoms (52). Pharmacological treatment of constipation can likewise be interpreted mechanistically. Osmotic agents increase luminal water, stimulant laxatives promote motility, secretagogues enhance intestinal secretion, and 5-HT4 agonists facilitate peristalsis (4, 7). Because these interventions alter transit and luminal conditions, their effects on microbial ecology and metabolite exposure warrant consideration, particularly when bowel dysfunction coexists with anorectal disease.

Microbiota and metabolite directed interventions offer additional opportunities but remain unevenly supported. In UC, FMT has shown efficacy in selected randomized trials of ulcerative colitis, although donor selection, treatment intensity, delivery route, and maintenance strategies remain heterogeneous (53, 54). Probiotics may improve some constipation outcomes, but responses depend on strain, dose, and baseline microbial composition (55). However, immune-directed treatment is already well established, including anti-TNF, anti-integrin, anti-IL-12/23 or anti-IL-23 agents, Janus kinase inhibitors, and sphingosine-1-phosphate receptor modulators (56). Treat-to-target strategies prioritize clinical remission and endoscopic healing, with increasing attention to histological and biomarker responses (21). Microbial and metabolic profiling may eventually refine this framework by identifying residual biological abnormalities that persist despite apparent mucosal improvement. Anal fistula requires a different therapeutic hierarchy because anatomical source control remains indispensable. Seton drainage, fistulotomy, advancement flap, ligation of the intersphincteric fistula tract, and other sphincter-preserving approaches are selected according to tract anatomy and continence risk. Crohn-associated fistulas often require combined medical and surgical treatment, and mesenchymal stromal cell therapy demonstrates that local immune modulation and regenerative repair can be therapeutically actionable (16, 57, 58). Perioperative microbiota management is less developed. Antibiotic stewardship, bowel optimization, wound hygiene, and control of active inflammation are currently more defensible than deliberate manipulation of local microbial communities, particularly in anorectal wounds continuously exposed to stool and perianal flora (59, 60).

A major methodological priority is to move beyond stool-only and cross-sectional designs. Stool does not necessarily represent rectal mucosa, the anal canal, hemorrhoidal tissue, abscess cavities, fistula tracts, perianal skin, or postoperative wounds, which differ substantially in oxygen exposure, epithelial phenotype, immune surveillance, trauma, and microbial contact. Paired fecal, mucosal, anorectal swab, lesion-specific tissue, and systemic metabolomic sampling should be used whenever feasible (11, 12, 27, 28, 50). Longitudinal sampling is equally important because molecular changes observed during active inflammation or tissue injury may be consequences rather than causes. Sampling before treatment, during healing, and after remission or recurrence would help distinguish stable predisposition signatures from transient responses. Phenotypic resolution is also essential. Constipation should be separated into relevant transit and defecatory subtypes; fistula cohorts should distinguish cryptoglandular from Crohn-associated, recurrent, or complex disease; hemorrhoidal cohorts should account for grade, prolapse, bleeding, thrombosis, and recurrence; and UC should be stratified by extent, activity, medication exposure, and degree of mucosal or histological healing. Without such granularity, molecular signatures may reflect mixed clinical states rather than mechanism-specific biology (17, 46, 47). Research increasingly implicates site-specific microbial communities, persistent inflammation, stromal remodeling, and defective repair, whereas hemorrhoidal disease remains supported mainly by clinical and structural evidence with limited microbiota-centered molecular data (1, 2, 11, 58, 60). The shared framework should therefore be interpreted as a set of overlapping biological modules rather than evidence that these disorders have equivalent pathogenesis.

Causal validation should be anchored to patient-derived signals. Organoids can test candidate metabolite effects on mucus production, epithelial junctions, inflammatory responses, and repair, while anaerobic co-cultures can examine microbial transformation of bile acids, tryptophan metabolites, and short-chain fatty acids. Tissue explants and fistula-derived cultures may preserve local immune-stromal interactions, and gnotobiotic or humanized models can test whether defined microbial communities transmit motility, barrier, or wound-healing phenotypes (17, 47, 61). Functional metabolite profiles may prove more transferable than individual taxonomic signatures because they map more directly onto host receptors and pathways, although diet, transit, collection, storage, and inflammatory activity must be standardized (33–41). Spatial and single-cell approaches are particularly relevant to fistula, where microbial persistence, epithelialization, immune-cell organization, fibroblast activation, and matrix remodeling occur within anatomically distinct tissue niches (61).

Prospective validation is necessary to prove clinical utility. Molecular signatures are valuable only when they outperform routine clinical variables in predicting outcomes or guiding treatment. In constipation, useful biomarkers could point to underlying mechanisms such as delayed transit, methane production, bile acid dysregulation, or pelvic floor dysfunction (7, 9, 10, 26, 55). In hemorrhoidal disease, the immediate value would lie in predicting recurrence or delayed healing (13–15, 19). In fistula, clinically useful signatures should improve prediction of nonhealing, recurrent abscess, or failure of definitive closure and could help identify patients requiring prolonged drainage, regenerative adjuncts, or closer surveillance (11, 12, 16, 61–63). In UC, molecular profiling may support relapse prediction, treatment selection, and treat-to-target monitoring (21, 56). Predictive models should undergo external validation, remain biologically interpretable, and demonstrate added value over clinical variables alone; for treatment or surgical decisions, calibration and decision-curve analysis are as important as discrimination (29, 32, 64).

Translation also depends on safety, standardized outcomes, and practical implementation. FMT and live biotherapeutics require rigorous donor screening, pathogen surveillance, and indication-specific risk assessment (53, 54), while metabolite or perioperative interventions must not compromise infection control or wound healing (59, 60). Medication exposure, diet, bowel preparation, antibiotics, laxatives, biologics, topical agents, fiber intake, hydration, and toileting behavior should be recorded because they can alter transit, microbial composition, metabolite production, epithelial physiology, and immune responses (10, 52). Outcome definitions likewise require harmonization. Constipation studies should report stool frequency, form, straining, incomplete evacuation, rescue medication use, and quality of life; hemorrhoidal studies should distinguish bleeding, prolapse, pain, recurrence, retreatment, and wound complications; fistula studies should separate clinical closure, radiological healing, drainage, recurrent abscess, continence, and patient-reported outcomes (62, 63); and UC studies should integrate endoscopic, histological, biochemical, and patient-reported endpoints (21).

The most realistic translational path is therefore not a universal microbiota-directed therapy, but a staged progression from well-phenotyped cohorts and anatomy-specific sampling to functional multiomics, causal validation, and mechanism-stratified intervention. Negative and inconsistent findings should remain visible, because constipation-associated microbial signatures vary with transit, diet, and medication, hemorrhoidal microbiota data remain sparse, fistula studies are frequently confounded by antibiotics and active sepsis, and UC signatures vary with activity, therapy, diet, and geography (5, 9, 11, 31, 64). Precision medicine is most likely to add value when it preserves these disease-specific distinctions while identifying shared, modifiable mechanisms that influence recurrence, healing, and treatment response.

## 7 Conclusions

Benign anorectal disorders and UC remain clinically distinct, yet they may share convergent biological features involving stool characteristics, microbial communities, metabolites, epithelial barrier function, immune activation, and tissue repair. Interpreting constipation, hemorrhoidal disease, anal fistula, and UC through potentially shared biological modules may generate sharper hypotheses than symptom-only or procedure-centered frameworks. The strongest current evidence derives from UC multi-omics and mucosal immunology, whereas anorectal disorders still require standardized molecular investigation. Priority directions include longitudinal sampling, site-specific anorectal microbiology, metabolomics, tissue-resolved immune and stromal profiling, and causal validation in experimental systems. When combined with rigorous phenotyping and external validation, these efforts may support recurrence prediction, perioperative optimization, and individualized adjunctive interventions across anorectal and colonic inflammatory disease. The principal contribution of this review is a cross-disease translational framework that connects anatomically distinct anorectal and colonic disorders through partially convergent molecular modules without implying biological equivalence. By integrating microbiota, metabolite, barrier, immune-stromal, and tissue-repair biology, the framework identifies testable molecular states that may transcend conventional diagnostic boundaries. Future anatomy-resolved, longitudinal, and causally validated multiomics studies can determine whether these shared states provide clinically useful biomarkers and therapeutic opportunities beyond disease-specific assessment alone.
